# CD2AP inhibits metastasis in gastric cancer by promoting cellular adhesion and cytoskeleton assembly

**DOI:** 10.1002/mc.23158

**Published:** 2020-01-28

**Authors:** Wangkai Xie, Chao Chen, Zheng Han, Jingjing Huang, Xin Liu, Hongjun Chen, Teming Zhang, Sian Chen, Chenbin Chen, Mingdong Lu, Xian Shen, Xiangyang Xue

**Affiliations:** ^1^ Department of General Surgery The Second Affiliated Hospital and Yuying Children's Hospital of Wenzhou Medical University Wenzhou China; ^2^ Department of Microbiology and Immunology, School of Basic Medical Science, Institute of Molecular Virology and Immunology, Institute of Tropical Medicine Wenzhou Medical University Wenzhou China; ^3^ Department of Oncology Wenzhou Hospital of Integrated Traditional Chinese and Western Medicine Affiliated to Zhejiang Chinese Medical University Wenzhou China

**Keywords:** CD2AP, cytoskeleton, gastric cancer, intercellular adhesion, invasion, migration

## Abstract

Diffuse gastric cancer (DGC) is a lethal malignancy lacking effective systemic therapy. Among the most provocative recent results in DGC has been that the alter of the cellular cytoskeleton and intercellular adhesion. CD2‐associated protein (CD2AP) is one of the critical proteins regulating cytoskeleton assembly and intercellular adhesion. However, no study has investigated the expression and biological significance of CD2AP in gastric cancer (GC) to date. Therefore, the aim of our study was to explore if the expression of CD2AP is associated with any clinical features of GC and to elucidate the underlying mechanism. Immunohistochemistry of 620 patient tissue samples indicated that the expression of CD2AP is downregulated in DGC. Moreover, a low CD2AP level was indicative of poor patient prognosis. In vitro, forced expression of CD2AP caused a significant decrease in the migration and invasion of GC cells, whereas depletion of CD2AP had the opposite effect. Immunofluorescence analysis indicated that CD2AP promoted cellular adhesion and influenced cell cytoskeleton assembly via interaction with the F‐actin capping protein CAPZA1. Overall, the upregulation of CD2AP could attenuate GC metastasis, suggesting CD2AP as a novel biomarker for the prognosis and treatment of patients with GC.

**Abbreviations**
CCK‐8: cell counting kit‐8
CD2AP: CD2‐associated protein
DGC: diffuse gastric cancer
EMT: epithelial‐mesenchymal transition
GC: gastric cancer
IGC: intestinal gastric cancer
IHC: immunohistochemistry
IOD: integrated optical density
TCGA: The Cancer Genome Atlas
TMA: the tissue microarray
TNM: tumor node metastasis

## 1 INTRODUCTION

Gastric cancer (GC) is one of the most prevalent cancers worldwide, especially in Asia, and is associated with a high mortality rate, with more than 700 000 people dying of the disease each year.^1^ Most cases of GC are diagnosed at a late stage characterized by metastasis. Even with the currently best treatment, the survival of GC patients with distant metastases is often poor.^2^, ^3^, ^4^ Despite great progress in identifying novel therapeutic targets for advanced or metastatic GC, the underlying molecular mechanisms are poorly understood, in part because of the biological heterogeneity of GC.^5^ However, accumulating evidence indicates that a reduction of intercellular adhesion and alterations in the cytoskeleton structure are the major causes of the development of GC.^6^ Moreover, several studies have shown that the intercellular adhesion‐related molecules such as E‐cadherin, ZO‐1, CAPZA1, and SCIN can impact the prognosis of cancer patients.^7^, ^8^, ^9^ Thus, identifying novel molecules or mutations involved in GC‐related intercellular adhesion or cytoskeleton assembly is an important step for understanding the pathogenesis of GC metastasis.

CD2‐associated protein (CD2AP) is an adhesion‐related adapter protein that plays an important role in the formation of epithelial cell junctions,^10^, ^11^, ^12^, ^13^ and has been reported as a key factor in the pathogenesis of foot‐related nephropathy.^14^, ^15^, ^16^, ^17^, ^18^ The ablation of CD2AP led to a disordered tissue structure in the lamina propria cells of the gastric mucosa, suggesting that CD2AP plays an important role in maintaining the normal morphological structure of the gastric mucosa.^19^ However, no study has investigated the expression and biological significance of CD2AP in GC to date. Therefore, in this study, we examined the expression of CD2AP in 564 GC clinical specimens and explored the role of CD2AP in the metastasis and proliferation of GC in vitro, with the aim of determining its potential application in the diagnosis and treatment of GC.

## 2 MATERIALS AND METHODS

### 2.1 Patients and follow‐up

We collected 564 gastric cancer tissue samples from patients who underwent surgical resection for GC at the Second Affiliated Hospital of Wenzhou Medical University (Wenzhou, China) from December 2006 to July 2011. The gastric adenocarcinoma and 56 paired adjacent normal tissue samples (at least 10 cm from the negative margin) were retrieved from the patients, fixed in formalin, and embedded in paraffin. The formalin‐fixed, paraffin‐embedded samples were confirmed by histopathological analysis.

The demographic and clinicopathological characteristics of the patients, including age, differentiation status, sex, serum carcinoembryonic antigen, Lauren type, levels of CA 72‐4 and CA19‐9, depth of invasion, lymph node metastasis, and tumor node metastasis (TNM) stage at the time of surgery (classified according to the American Joint Committee on *Cancer Staging Manual*, 8th edition), were assessed. The patients comprised 402 males and 162 females ranging in age from 20 to 86 years (median, 59 years). The study was approved by the Review Board of the Second Affiliated Hospital of Wenzhou Medical University. The tissue microarray (TMA) was constructed as described previously.^20^ All the patients were aware of the research and signed the informed consent form.

### 2.2 Immunohistochemistry

Immunohistochemistry on a tissue microarray, constructed as described previously,^20^ was performed for the detection of CD2AP expression. In brief, the sections were dewaxed by incubation with dimethyl benzene at 45°C for 60 minutes and then immersed in distilled water. Endogenous peroxidase activity was inhibited by incubation in a 0.5% hydrogen peroxide bath for 10 minutes. After washing three times with 0.01 M phosphate‐buffered saline (PBS, pH 7.4), the slides were immersed in citrate antigen retrieval buffer (Zhongshan Golden Bridge Biotechnology, Beijing, China). After blocking with sheep serum for 30 minutes, the sections were incubated with anti‐CD2AP antibody (sc‐25272; Santa Cruz Biotechnology, Dallas, TX; 1:50 dilution; Figure S1B) in a humidified chamber at 24°C for 2 hours. After washing three times with PBS, a Dako EnVision FLEX detection system (Dako, Carpinteria, CA) was used for visualization, according to the manufacturer's instructions. The sections were counterstained with hematoxylin, dehydrated, and sealed with neutral gum.

### 2.3 Quantification of CD2AP expression

The TMA was obtained using a digital slice scanner for the Whole Slide Image (Easyscan6; MOTIC Medical Diagnostic Systems, Fuzhou, China) and separated into single spots. Microarray spots with no tumor tissue, missing spots, and spots with the minimal valid area were then eliminated. The tumor‐positive area was selected and the integrated optical density (IOD) of CD2AP was determined using Image Pro Plus 6 (IPP; Media Cybernetics, Rockville, MD). Finally, the CD2AP score (IOD/tumor‐positive area) was calculated.

### 2.4 Bioinformatics analysis

The Cancer Genome Atlas (TCGA) GC data were downloaded from https://tcga-data.nci.nih.gov/docs/publications/tcga. The data set contains survival data, clinical information, and messenger RNA (mRNA) expression levels. For validation, we obtained four independent microarray data sets (GSE26942, GSE2669, GSE15460, and GSE62254) containing cancer and paracancerous samples, and another four independent microarray data sets (GSE14208, GSE15459, GSE57303, and GSE62254) with prognostic information from GEO. The mRNA levels of the validation set were measured by the Affymetrix Human Genome U133 Plus 2.0 Array (Thermo Fisher, Massachusetts, CA) on a log2 scale. Gene expression in the discovery set was determined using the Illumina HiSeq platform and transformed to the log2 scale. According to the publication guidelines, the data sets may be used for publication without restriction or limitation (https://cancergenome.nih.gov/publications/publicationguidelines, https://www.ncbi.nlm.nih.gov/geo/info/disclaimer.html).

### 2.5 Cell lines

Two GC cell lines, MGC‐803 and BGC‐823, were purchased from the Cell Bank of the Chinese Academy of Sciences (Shanghai, China). The cells were maintained in Dulbecco's modified Eagle's medium (Gibco, Grand Island, NY) supplemented with 10% heat‐inactivated fetal bovine serum (FBS; Gibco).

### 2.6 CD2AP knockdown by small interfering RNA (siRNA)

Two siRNA oligos against CD2AP and CAPZA1 were designed (siCD2AP‐1: 5′‐GTG GAA CCC TGA ATA ACA A‐3′/siCD2AP‐2: 5′‐ GGG CGA ACT TAA TGG TAA A‐3′, and siCAPZA1: 5′‐CUG UGA AGA UAG AAG GAU‐3′)^21^ and synthesized by RiboBio (Guangzhou, China). The siRNAs were transfected into GC cells at a final concentration of 20 nM using Lipofectamine 2000 reagent (Invitrogen, Carlsbad, CA).

### 2.7 Construction of plasmids expressing CD2AP

The DNA fragment carrying the *CD2AP* open reading frame with an HA tag was inserted into the pcDNA3.1(+) vector at the *BamH*I and *Xho*I restriction sites. The constructed plasmid was sequenced for confirmation. The plasmid was then transfected into BGC‐823 and MGC‐803 GC cells using Lipofectamine 2000, respectively, according to the manufacturer's protocol. The expression of CD2AP in these cells was confirmed using both HA‐tag and CD2AP‐specific antibodies.

The HA‐tagged *CD2AP* DNA fragment was inserted into the pENTR3C vector at the *Xho*I and *EcoR*I restriction sites. This fragment was then transferred into the pInducer20 vector using Gateway cloning technology (Invitrogen). Lentiviral particles were produced in HEK293T cells, using the Lenti‐XTM HTX Packaging System (Hanbio Biotechnology, Shanghai, China), titrated onto MGC‐803 cells, and selected by puromycin (1 µg/mL; Sigma‐Aldrich, St. Louis, MO) to achieve optimal overexpression of the CD2AP protein at a minimal viral load, resulting in the MGC‐803‐CD2AP cell line. We inserted the CDS of CD2AP behind a tetracycline‐responsive promoter, called TET‐on system. Doxycycline (DOX; Sigma‐Aldrich) a type of tetracycline, used in this study to activate the tetracycline promoter. Only when cultured with DOX can the MGC‐803‐CD2AP cells express CD2AP. MGC‐803‐CD2AP cells without DOX were used as control. We have added these comments in the revised manuscript in the part of Materials and methods, Construction of the plasmids for expressing CD2AP.

### 2.8 Western blot analysis

Total protein was extracted from the cells using RIPA lysis buffer (Beyotime, Haimen, China) supplemented with a protease inhibitor cocktail (Sigma‐Aldrich). Equal quantities of protein samples were separated on a sodium dodecyl sulfate‐polyacrylamide gel and transferred onto a polyvinylidene fluoride membrane (Bio‐Rad, Hercules, CA). The membrane was blocked with 5% skimmed milk in Tris‐buffered saline containing Tween 20 at room temperature (25℃) for 1 hour, and then probed overnight at 4°C with an antibody against either CD2AP (dilution 1:1000), CAPZ (dilution 1:1000; Cell Signaling Technology, Danvers, MA), or GAPDH (dilution 1:1000; Cell Signaling Technology) overnight at 4°C. Subsequently, the membranes were exposed to horseradish peroxidase‐labeled IgG for 2 hours and the bands were visualized using a Bio‐Rad imaging system.

### 2.9 Migration and invasion assays

Twenty‐four‐well Transwell chambers with 8‐mm pore‐size membranes were used to examine the migration and invasion abilities of the transfected GC cells. In the invasion assay, the chambers were coated with Matrigel (BD Pharmingen, San Jose, CA). The two transfected GC cell lines (BGC‐823 and MGC‐803; 1 × 10^5^ cells) were seeded into the upper chamber in serum‐free medium, respectively. Thereafter, the medium containing 10% FBS was added to the lower chamber as a chemoattractant. After incubating for 24 hours, the cells above the Matrigel layer were removed and the cells below the membrane were fixed with ice‐cold methanol, stained with crystal violet, and counted under a microscope (Leica, London, UK) from five randomly chosen fields per well.

### 2.10 Cell viability

Cell viability was assessed using cell counting kit‐8 (CCK‐8) (Dojindo, Kumamoto, Japan). The two GC cell lines were seeded in 96‐well plates (5 × 10^3^ cells/well), respectively, and transfected with either vectors (pcDNA3.1, pcDNA3.1‐CD2AP‐HA) or siRNA oligos (siNC, siCD2AP‐2, siCD2AP‐3). After 48 hours, the cells were treated with CCK‐8 reagent for 2 hours at 37°C. The absorbance at 450 nm was measured using a microplate reader. All experiments were carried out in triplicate.

### 2.11 Immunofluorescence

Cell focal adhesion was detected by immunofluorescence. In brief, the expression of CD2AP was induced in MGC‐803‐CD2AP cells (2 × 10^5^ cells/well in six‐well plates containing glass coverslips) by treatment of DOX (1 µg/mL) for 48 hours. The cells were then fixed in 4% paraformaldehyde at 37°C for 10 minutes and permeabilized with 0.1% Triton X‐100 at 37°C for 10 minutes. After blocking with 10% goat serum (Beyotime) in PBS for 60 minutes at 37°C, the cells were incubated with a rabbit antibody specific to zyxin (Abcam, Cambridge, UK) and DyLight488‐conjugated goat antirabbit antibody in PBS supplemented with 10% goat serum for 1 hour at 37°C. The cells were subsequently incubated with Hoechst 33258 (Beyotime) for 5 minutes at room temperature. The coverslips were mounted and the signals were visualized under a fluorescence microscope (Carl Zeiss, Jena, Germany).

### 2.12 Co‐immunoprecipitation

Two micrograms of plasmid DNA [either pcDNA3.1(+)‐CD2AP‐HA or pcDNA3.1(+)] were transfected into MGC‐803 cells using Lipofectamine 2000, according to the manufacturer's instructions. After 48 hours, the cells were lysed for 10 minutes on ice with NP40 buffer (Beyotime). The lysates were centrifuged at 12,000*g* for 10 minutes at 4°C. The supernatant (1 mL) was mixed with 25 μL of anti‐HA magnetic beads (Invitrogen) and incubated at room temperature for 30 minutes with mixing. The beads were collected using a magnetic stand. The protein was eluted with the lane marker nonreducing sample buffer (Invitrogen) at 95°C for 10 minutes, and then analyzed by immunoblotting.

### 2.13 Adhesion assay

MGC‐803‐CD2AP cells were pretreated with 1 µg/mL of DOX for 48 hours, and incubated in a 24‐well plate precoated with 2 μg/ml human fibronectin (R&D Systems, Minneapolis, MN) for 2 hours at 37°C. The fibronectin was removed, and the cells were washed with PBS three times. The pretreated cells were then seeded in 24‐well plates (1 × 10^3^ cells/well) and incubated for 1 hour at 37°C. The nonadherent cells were washed off with PBS three times, and the remaining adherent cells were fixed with ice‐cold methanol, stained with crystal violet, and counted under a microscope. Three representative fields were randomly counted for analysis.

### 2.14 Statistical analysis

All statistical analyses were performed using the SPSS 17.0 software package (IBM, Chicago, IL). Data are reported as means ± SEM. Analysis of variance and independent‐sample *t* tests were performed to assess the differences between groups. The cutoff value of CD2AP expression in GC was determined by the Youden index based on the overall survival‐specific receiver operating curve. The CD2AP immunohistochemical scores and the CD2AP mRNA levels in GEO databases and TCGA database were then divided into high‐expression and low‐expression groups. Univariate survival analysis and Kaplan‐Meier's analysis with a log‐rank test were performed to construct survival curves. A value of *P* < .05 was considered statistically significant.

## 3 RESULTS

### 3.1 The expression level of CD2AP is significantly lower in diffuse GC

**Figure 1:**
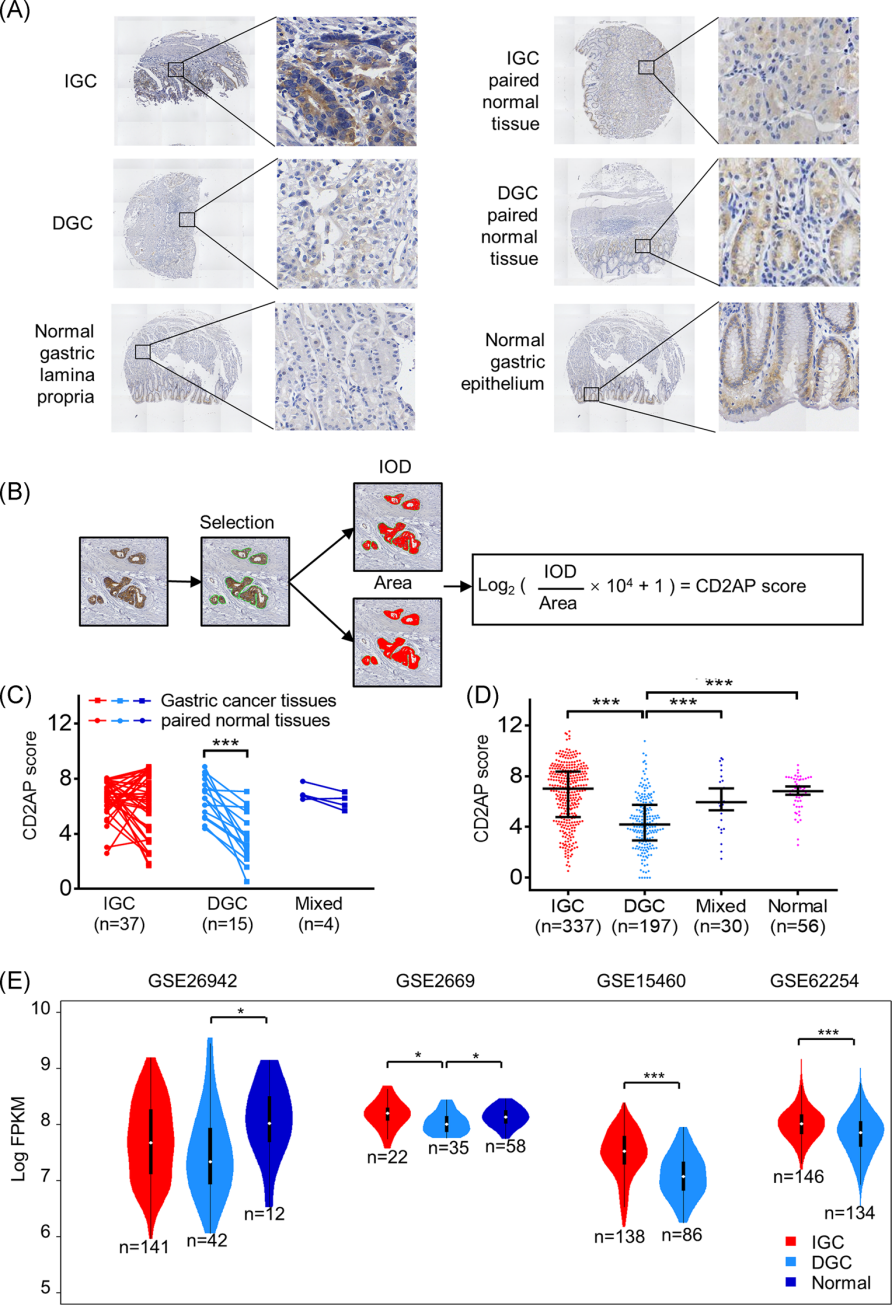
The expression of CD2AP is significantly lower in diffuse gastric cancer. A, Immunohistochemical expression of CD2AP in IGC, DGC, paired normal tissue, normal gastric lamina propria, and normal gastric epithelium. B, Select the gland region by IPP, analyze the IOD of the CD2AP positive region and the total area of the gland, and calculate the CD2AP score. C, In the paired specimens, the expression of CD2AP in DGC tissues (*n* = 15) was significantly lower than that in the matched normal gastric mucosa (***P < .001), but not in IGC (*n* = 37) and mixed gastric cancer (*n* = 4) (*P* > .05). D, The expression of CD2AP in DGC is significantly lower than that in IGC, mixed type and normal gastric mucosa (***P < .001). E, Violin picture show CD2AP expression in DGC, IGC, and normal gastric tissue. For each Violin picture, median and ranges are indicated (*P < .05, ***P < .001). CD2AP, CD2‐associated protein; DGC, Diffuse gastric cancer; IGC, intestinal gastric cancer; IOD, integrated optical density, IPP, Image Pro Plus 6 [Color figure can be viewed at wileyonlinelibrary.com]

We first used immunohistochemistry to examine the expression of CD2AP in the tissues from 564 patients with GC, including 56 adjacent noncancerous tissues. CD2AP was mainly expressed in the cytoplasm of the cancer cells, with some expression detected in the nucleus. Only a small amount of CD2AP was expressed in the stromal cells. In the normal gastric mucosa, CD2AP was predominantly expressed in the mucosal epithelial layer (Figure 1A). The scores of CD2AP expression are shown in Figure 1B.

Based on tissue structures and biological behaviors, GC can be categorized into intestinal gastric cancer (IGC) and diffuse gastric cancer (DGC).^22^ The expression of CD2AP in these two tissue types was significantly distinct. In the paired tissue samples, the expression of CD2AP in DGC tissue (*n* = 15) was significantly lower than that in the paired normal tissues (*P* < .001); however, there was no significant difference in CD2AP expression scores between IGC (*n* = 37) and mixed GC (*n* = 4) tissues (*P* > .05) (Figure 1C). Moreover, the 620 tissue samples were divided into IGC (*n* = 337), DGC (*n* = 197), mixed GC (*n* = 30), and normal gastric mucosa (*n* = 56). The expression of CD2AP in DGC was significantly lower than that in IGC, mixed type, or normal gastric mucosa (*P* < .001), but there was no significant difference between that in IGC, mixed GC, and normal gastric mucosa (*P* > .05) (Figure 1D). Similarly, in the GEO data sets (GSE26942, GSE2669, GSE15460, and GSE62254), the expression of CD2AP in DGC was lower than that in IGC or normal gastric mucosa, but there was no significant difference in CD2AP expression between IGC and the normal gastric mucosa in these four data sets (Figure 1E).

### 3.2 GC patients with a low expression level of CD2AP have a poor prognosis

**Figure 2:**
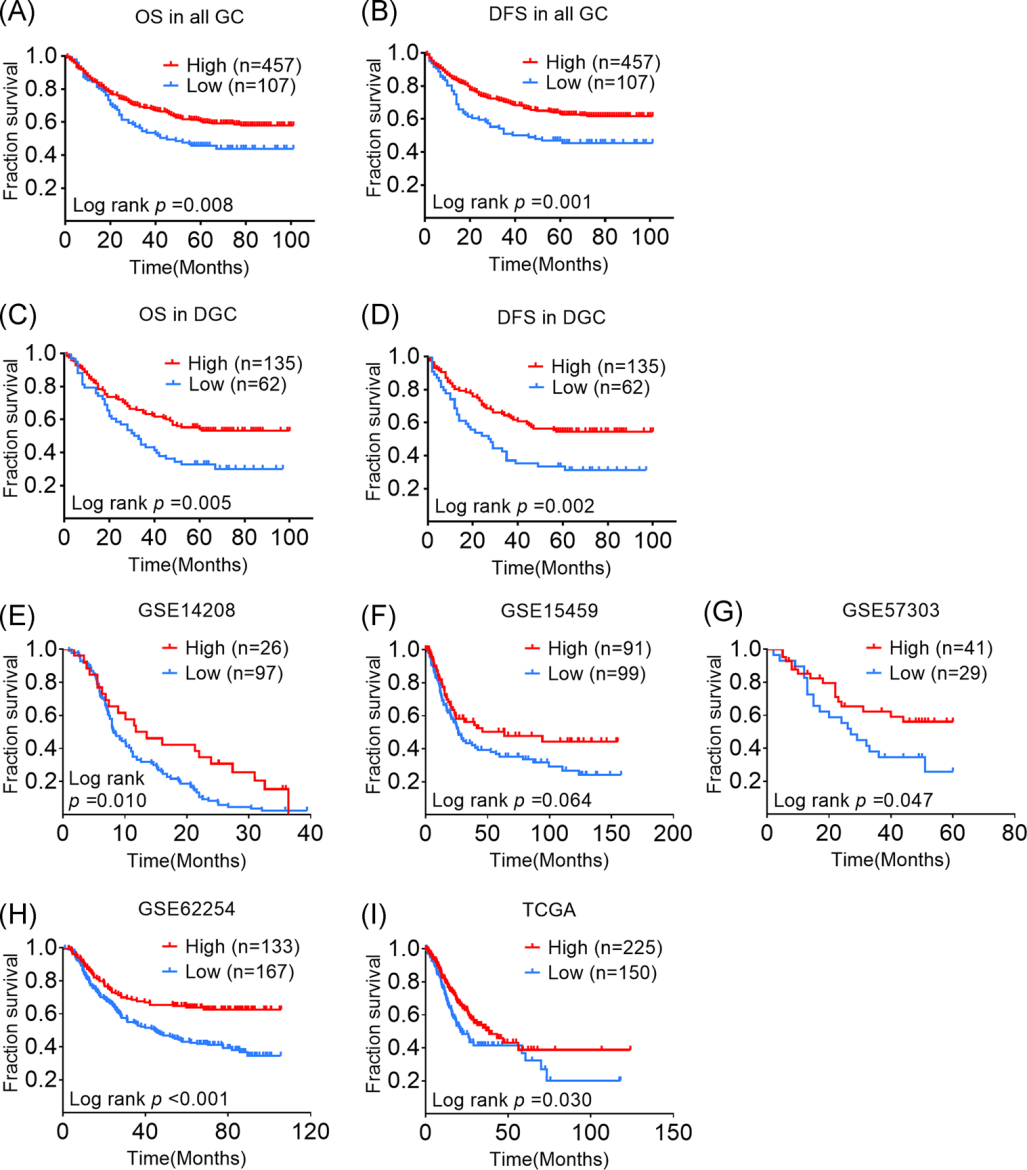
GC patients with a low expression level of CD2AP have a poor prognosis. A and B, Low CD2AP expression in 564 gastric cancer predicts poor OS times and DFS times. C and D, Low CD2AP protein expression in 187 DGC predicts poor OS times and DFS times. E‐H, Low CD2AP protein expression in GED data sets (GSE14208, GSE15459, GSE57303, GSE62254) predicts poor OS times. I, Low CD2AP protein expression in 375 TCGA gastric cancer data sets predicts poor OS times. CD2AP, CD2‐associated protein; DGC, diffuse gastric cancer; DFS, disease‐free survival; GC, gastric cancer; OS, overall survival; TCGA, The Cancer Genome Atlas [Color figure can be viewed at wileyonlinelibrary.com]

To investigate the effect of CD2AP expression on patients with GC, we analyzed the relationship between CD2AP expression and clinical features. There was a statistically significant difference between the patients with high and low CD2AP expression according to T stage (*P* = .044), N stage (*P* = .038), TNM stage (*P* = .041), differentiation status (*P* = .001), Lauren type (*P* < .001), Bowman's type (*P* = .022), and WHO pathological type (*P* < .001), regardless of sex, age, long diameter of the mass, and distant metastasis (Table 1). The Kaplan‐Meier survival analysis showed that the group with lower CD2AP expression had a worse overall survival (OS) rate (Figure 2A) and worse disease‐free survival (DFS) rate (Figure 2B). Considering that the expression of CD2AP in DGC was significantly lower than that in the paired normal tissues and that the expression of CD2AP in IGC was not significantly different from that in the paired normal tissues, we further analyzed CD2AP expression in DGC and found that CD2AP was an independent protective factor for overall survival (*P* = .021, odds ratio [OR] = 0.547, 95% confidence interval [CI] = 0.328‐0.912) as well as for disease‐free survival (*P* = .003, OR = 0.460, 95% CI = 0.276‐0.766) in DGC (Figure 2C,D and Table 2). In addition, a low level of CD2AP expression also indicated a poor prognosis in the GSE14208 (*P* = .010), GSE57303 (*P* = .047), GSE62254 (*P* < .001), and TCGA (*P* = .030) databases. This trend was also present in the GSE15459 data set, but the difference was not statistically significant (*P* = .064) (Figure 2E‐I). These results suggest that CD2AP may act as a tumor suppressor in GC.

**Table 1:** Clinicopathological features and CD2AP in GC

Clinicopathological features	Low (*n* = 107, 19.0%)	High (*n* = 45, 81.0%)	*P*‐value
Sex			.681
Male/female	78 (72.9%)/29 (27.1%)	324 (70.9%)/133 (29.1%)	
Age^a^			.586
<59/≥59	52 (48.6%)/55 (51.4%)	206 (45.7%)/245 (54.3%)	
Tumor size^a^			.131
<4 cm/≥4 cm	40 (37.7%)/66 (62.3%)	209 (45.8%)/247 (54.2%)	
Invasion depth			.044*
T1 + T2/T3 + T4	28 (26.4%)/78 (73.6%)	168 (36.8%)/289 (63.2%)	
Lymphatic metastasis			.038*
N0 + N1/N2 + N3	45 (43.3%)/59 (56.7%)	240 (54.5%)/200 (45.5%)	
Distant metastases			.442
			.752^b^
No/yes	105 (98.1%)/2 (1.9%)	442 (96.7%)/15 (3.3%)	
TNM			.041*
I + II/III + IV	39 (37.5%)/65 (62.5%)	214 (48.6%)/226 (51.4%)	
Differentiation			.001*
Moderate + Well/poor	34 (35.4%)/62 (64.6%)	232 (53.7%)/200 (46.3%)	
Lauren type			<.001^b^ ^,^ *
Intestinal	42 (39.3%)	295 (64.6%)	
Diffuse	62 (57.9%)	135 (29.5%)	
Mixed	3 (2.8%)	27 (5.9%)	
Borrmann type			.022^b^ ^,^ *
I	11 (10.7%)	49 (11.7%)	
II	21 (20.4%)	140 (33.4%)	
III	66 (64.1%)	200 (47.7%)	
IV	5 (4.9%)	30 (7.2%)	
WHO pathological type			<.001^b^ ^,^ *
TA	33 (30.8%)	253 (55.4%)	
PA	2 (1.9%)	6 (1.3%)	
MA	10 (9.3%)	6 (1.3%)	
PCC	34 (31.8%)	87 (19%)	
MC	28 (26.2%)	105 (23.0%)	
Recurrence			.002*
No/yes	45 (44.1%)/57 (55.9%)	260 (60.7%)/168 (39.3%)	

Abbreviations: CD2AP, CD2‐associated protein; GC, gastric cancer; MA, mucinous adenocarcinoma; MC, mixed carcinoma; PA, papillary adenocarcinoma; PCC, poorly cohesive carcinoma; TA, tubular adenocarcinoma; TNM, tumor node metastasis; WHO, World Health Organization.

^a^ Divided into two groups by the median.

^b^ Calculated using Fisher's exact test.

^c^ A small number of patient's clinical information was missing.

* Statistically significant (*P* < .05).

**Table 2:** Association of CD2AP protein expression and clinicopathological factors with OS and DFS in the GC cohort using Cox regression analysis

	OS univariate analysis		OS multivariate analysis		DFS univariate analysis		DFS disease‐free survival	
Clinicopathological features	OR (95% CI)	*P*‐value	OR (95% CI)	*P*‐value	OR (95% CI)	*P*‐value	OR (95% CI)	*P*‐value
CD2AP (high vs low)	0.666 (0.492‐0.900)	.008*	0.547 (0.328‐0.912)	.021*	0.656 (0.484‐0.890)	.007*	0.460 (0.276‐0.766)	.003*
Sex (female vs male)	1.038 (0.794‐1.357)	.785	1.098 (0.636‐1.894)	.738	1.047 (0.797‐1.374)	.743	1.263 (0.733‐2.177)	.401
Age^a^ (≥59 vs <59)	1.342 (1.054‐1.709)	.017*	1.331 (0.854‐2.074)	.207	1.348 (1.054‐1.723)	.017*	0.980 (0.618‐1.553)	.931
Tumor size^a^ (≥4 cm vs <4 cm)	4.269 (3.213‐5.673)	.001*	1.157 (0.600‐2.233)	.663	4.009 (3.007‐5.344)	.001*	1.316 (0.678‐2.554)	.417
Invasion depth (T3 + T4 vs T1 + T2)	2.106 (1.837‐2.416)	.001*	2.503 (1.021‐6.133)	.045*	6.188 (4.292‐8922)	.001*	2.873 (1.139‐7.247)	.025*
Lymphatic metastasis (N2 + N3 vs N0 + N1)	2.034 (1.804‐2.292)	.001*	2.696 (1.136‐6.400)	.025*	4.127 (3.083‐5.523)	.001*	2.715 (1.156‐6.376)	.022*
Distant metastases (yes vs no)	6.250 (3.883‐10.059)	.001*	1.474 (0.347‐6.262)	.599	5.447 (3.269‐9.075)	.001*	0.278 (0.045‐1.706)	.167
TNM (III + IV vs I + II)	3.523 (2.856‐4.346)	.001*	1.855 (0.705‐4.883)	.211	5.627 (4.031‐7.856)	.001*	1.897 (0.720‐4.997)	.195
Differentiation (poorly vs moderately + well)	0.688 (0.548‐0.864)	.001*	1.245 (0.599‐2.590)	.557	0.673 (0.525‐0.861)	.002*	1.200 (0.559‐2.576)	.64
WHO pathological type (PA vs TA)	1.094 (0.347‐3.441)	.879	0.228 (0.06‐0.8630)	.029*	0.818 (0.531‐1.259)	.36	0.418 (0.119‐1.467)	.173
WHO pathological type (MA vs TA)	0.821 (0.361‐1.866)	.638	0.692 (0.334‐1.434)	.322	0.927 (0.280‐3.070)	.901	0.909 (0.407‐2.027)	.815
WHO pathological type (PCC vs TA)	1.462 (1.067‐2.002)	.018*	0.348 (0.129‐0.940)	.037*	0.813 (0.352‐1.880)	.629	0.392 (0.132‐1.165)	.092
WHO pathological type (MC vs TA)	0.684 (0.425‐1.100)	.117	0.353 (0.143‐0.870)	.024*	1.218 (0.767‐1.935)	.402	0.423 (0.160‐1.115)	.082
Borrmann type (II vs I)	0.173 (0.096‐0.311)	.001*	0.823 (0.314‐2.158)	.692	0.158 (0.087‐0.287)	.001*	0.665 (0.249‐1.777)	.416
Borrmann type (III vs I)	0.223 (0.142‐0.350)	.001*	0.762 (0.288‐2.016)	.584	0.209 (0.132‐0.287)	.001*	0.681 (0.260‐1.789)	.436
Borrmann type (IV vs I)	0.531 (0.357‐0.788)	.001*	1.746 (0.532‐5.734)	.358	0.493 (0.330‐0.739)	.001*	3.076 (0.752‐12.584)	.118

Abbreviations: CD2AP, CD2‐associated protein; CI, confidence interval; DGC, diffuse gastric cancer; MA, mucinous adenocarcinoma; MC, mixed carcinoma; PA, papillary adenocarcinoma; OR, odds ratio; PCC, poorly cohesive carcinoma; TA, tubular adenocarcinoma; WHO, World Health Organization.

^a^ Divided into two groups by the median.

* Statistically significant (*P* < .05).

### 3.3 Modulation of CD2AP expression alters cell migration and invasion

**Figure 3:**
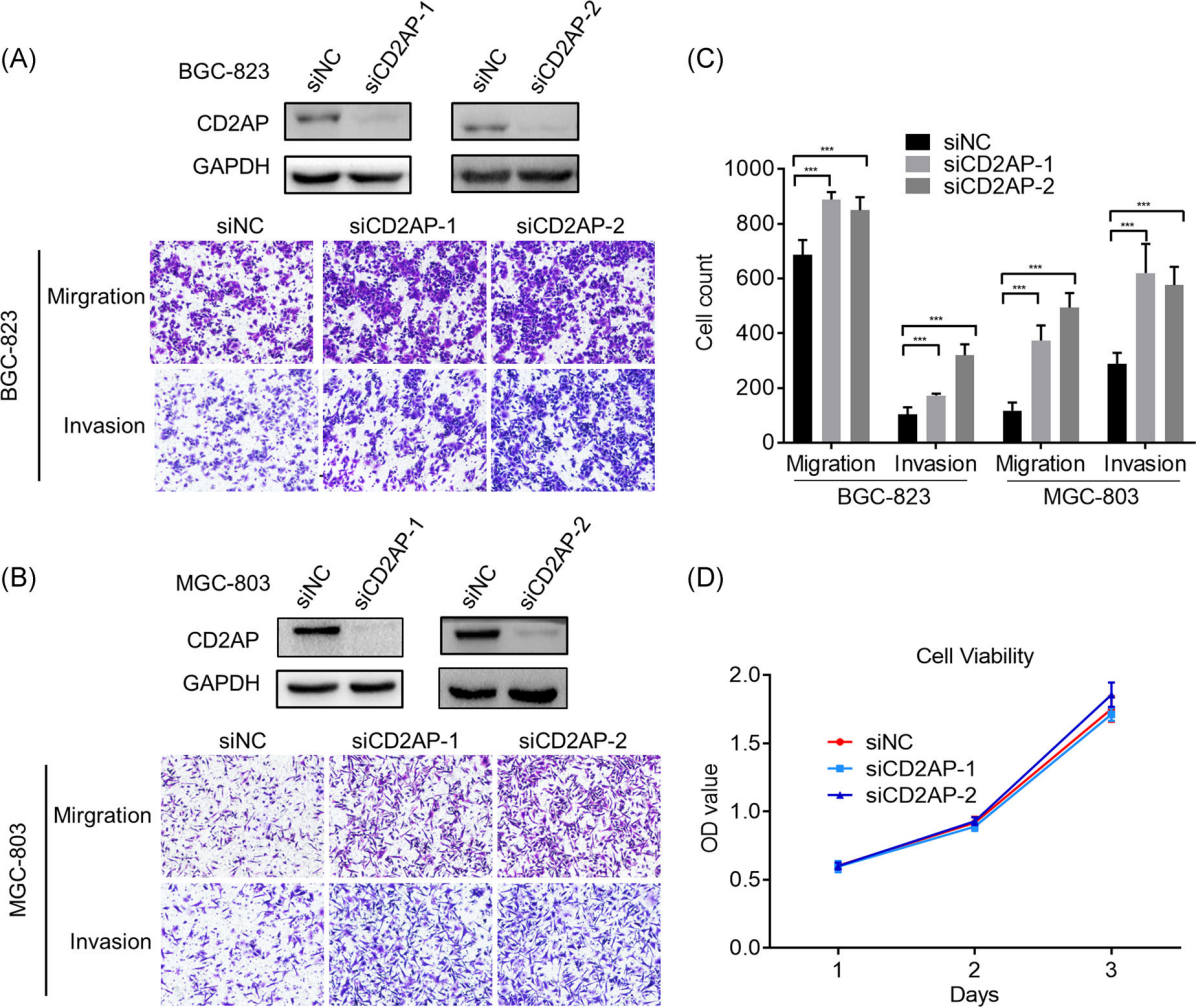
Depletion of CD2AP expression enhanced gastric cancer cell migration and invasion. A and B, The specificity of the two different CD2AP siRNAs in downregulating CD2AP gene expression. BGC‐823, MGC‐803 transfected with empty (siNC) or CD2AP siRNAs (si‐CD2AP‐1, si‐CD2AP‐2) were analyzed by immunoblotting with an antibody to CD2AP. An antibody to GAPDH was used as an equal loading control. The effect of CD2AP depletion on in vitro migration *and* invasion ability of BGC‐823, MGC‐803 cells. C, The migration rate and invasion rates of CD2AP‐overexpressing BGC‐823, MGC‐803 cell lines were markedly increased compared with that of the control cells (***P < .001). D, The proliferation rates of the CD2AP‐depleting BGC‐823 cells were not significantly different from those of control cells. CD2AP, CD2‐associated protein; GAPDH, glyceraldehyde 3‐phosphate dehydrogenase; siRNA, small interfering RNA [Color figure can be viewed at wileyonlinelibrary.com]

**Figure 4:**
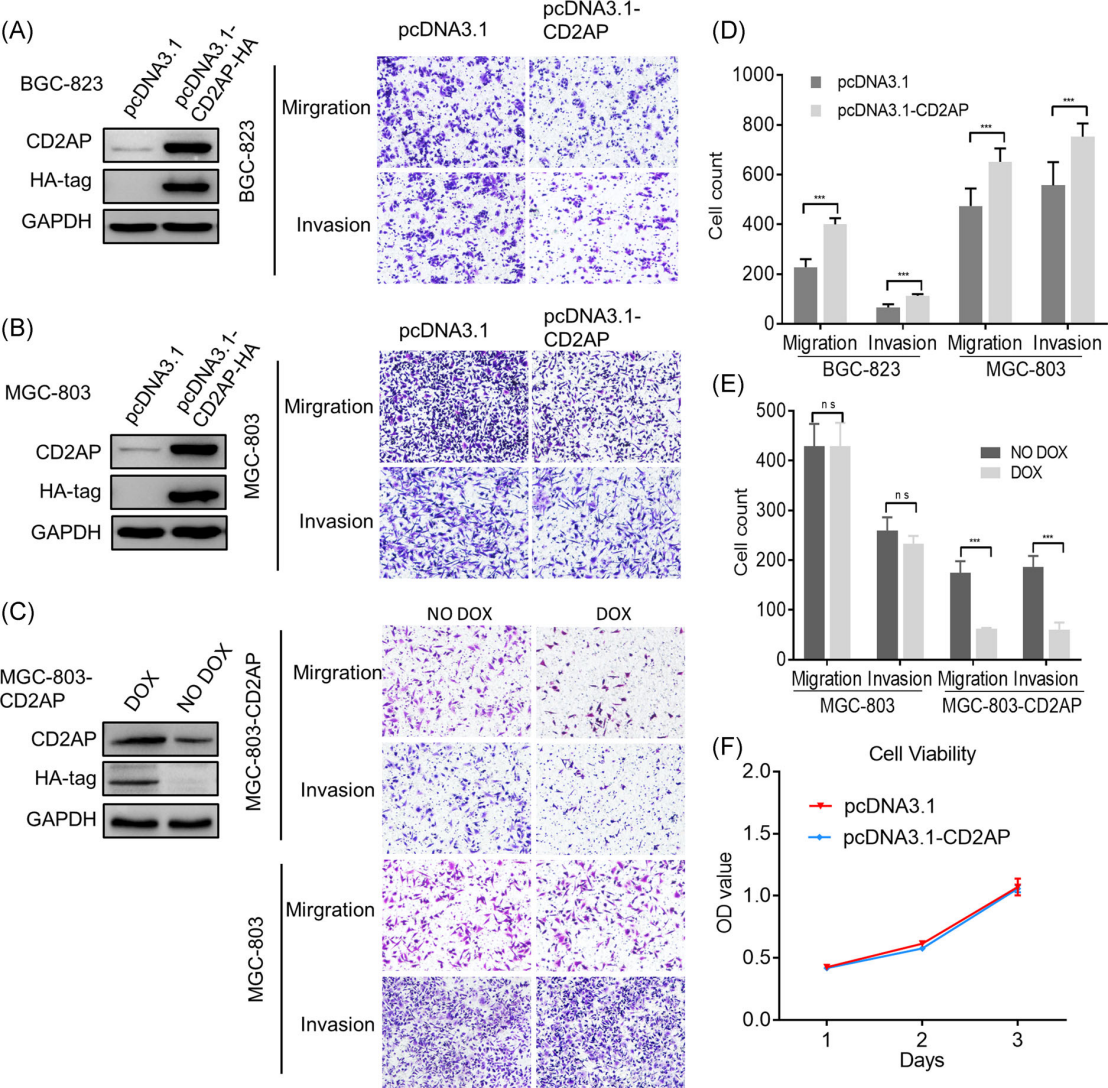
Overexpression of CD2AP inhibition gastric cancer cell migration and invasion. A‐C, BGC‐823, MGC‐803 transfected with pcDNA3.1 and pcDNA3.1‐CD2AP‐HA upregulated CD2AP gene expression, MGC‐803‐CD2AP induction CD2AP expression by 1 µg/mL DOX, and were analyzed by immunoblotting with an antibody to CD2AP. An antibody to GAPDH was used as an equal loading control. The effect of CD2AP‐overexpressing depletion on in vitro migration and invasion ability of BGC‐823, MGC‐803 cell lines. D and E, The migration rate and invasion rates of CD2AP‐depleting BGC‐823, MGC‐803, MGC‐803‐CD2AP cell lines were markedly increased compared with that of the control cells. (***P < .001). F, The proliferation rates of the CD2AP‐overexpressing BGC‐823 cells were not significantly different from those of control cells. CD2AP, CD2‐associated protein; DOX, doxycycline; GAPDH, glyceraldehyde 3‐phosphate dehydrogenase [Color figure can be viewed at wileyonlinelibrary.com]

To examine how CD2AP influences the function of GC cells in vitro, we depleted CD2AP in BGC‐823 and MGC‐803 GC cells by siRNA (Figure S2A), respectively. The results from Transwell assays showed that the depletion of CD2AP by both siRNAs significantly enhanced the migration and invasion of both GC cell lines (Figure 3A‐C). By contrast, transient overexpression of CD2AP in both BGC‐823 and MGC‐803 GC cells by transfection of pcDNA3.1‐CD2AP resulted in significant inhibition of cell migration and invasion (Figures 4A,B and 4D). Similar results were observed from the stable overexpression of CD2AP induced by DOX in the MGC‐803‐CD2AP cells (Figures 4C and 4E). Cell proliferation analyses indicated that neither knockdown nor overexpression of CD2AP impact the viability of BGC‐823 GC cells (Figures 3D and 4E).

### 3.4 Overexpression of CD2AP promoted cellular adhesion and cytoskeleton assembly in GC cells

**Figure 5:**
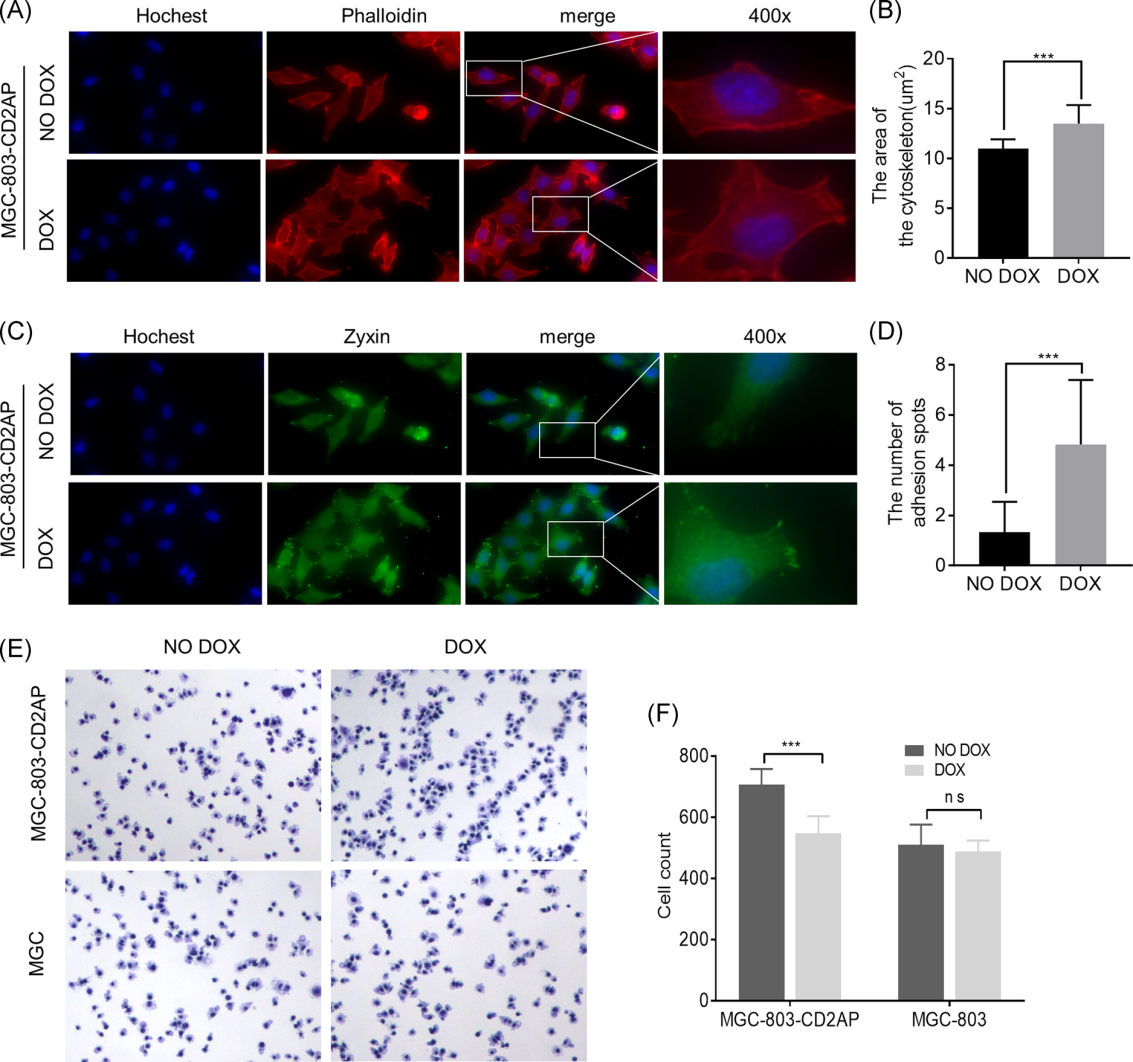
Overexpression of CD2AP promoted cellular adhesion and cytoskeleton assembly in gastric cancer cells. A, MGC‐803‐CD2AP cell induced express by 1 µg/mL DOX for 48 hours, the cytoskeleton was labeled with Phalloidin. B, The average area of the cytoskeleton is significantly enhanced in CD2AP overexpression MGC‐803‐CD2AP cell (***P < .001). C, MGC‐803‐CD2AP cell induced express by 1 µg/mL DOX for 48 hours, cell adhesion spots were labeled with zyxin antibody. D, The number of adhesion spots in cell is significantly increased in CD2AP overexpression MGC‐803‐CD2AP cell (***P < .001). E and F, MGC‐803‐CD2AP cell induced express by 1 µg/mL DOX for 48 hours, overexpress CD2AP significantly increase the adhesion ability of GC cells (***P < .001). CD2AP, CD2‐associated protein; DOX, doxycycline; GC, gastric cancer [Color figure can be viewed at wileyonlinelibrary.com]

CD2AP is an adhesion‐related adapter protein that plays an important role in the formation of epithelial cell junctions.^23^ The process of GC cell migration includes the extension of flaky pseudopods, formation of focal adhesions, and deagglomeration of tail adhesion spots.^24^ Many studies have shown that both intercellular adhesion and cytoskeleton proteins regulate cell migration by affecting the epithelial‐mesenchymal transition (EMT).^25^, ^26^ However, our results indicated that altered expression of CD2AP did not impact the expression of EMT‐related markers such as E‐cadherin and vimentin (Figure S3). Next, we induced the expression of CD2AP in MGC‐803‐CD2AP cells by DOX. Immunofluorescence analyses with phalloidin labeling showed that overexpression of CD2AP enhanced the assembly of cytoskeleton F‐actins compared with that of the cells without treatment of DOX (Figure 5A, *P* < .01). Moreover, as zyxin is a LIM domain protein localized mainly at focal adhesion plaques,^27^ we used an antibody against zyxin as a marker to detect cell adhesion spots. The number of focal adhesion spots was significantly increased in MGC‐803‐CD2AP cells treated with DOX when compared to that of the cells without DOX treatment (Figure 5B, *P* < .01). Moreover, the cell adhesion ability was significantly increased in MGC‐803‐CD2AP cells treated with DOX compared with that of the cells without treatment of DOX (Figure 5A, *P* < .01).

### 3.5 The interaction of CD2AP with CAPZA1 mediates cellular adhesion and cytoskeleton assembly

**Figure 6:**
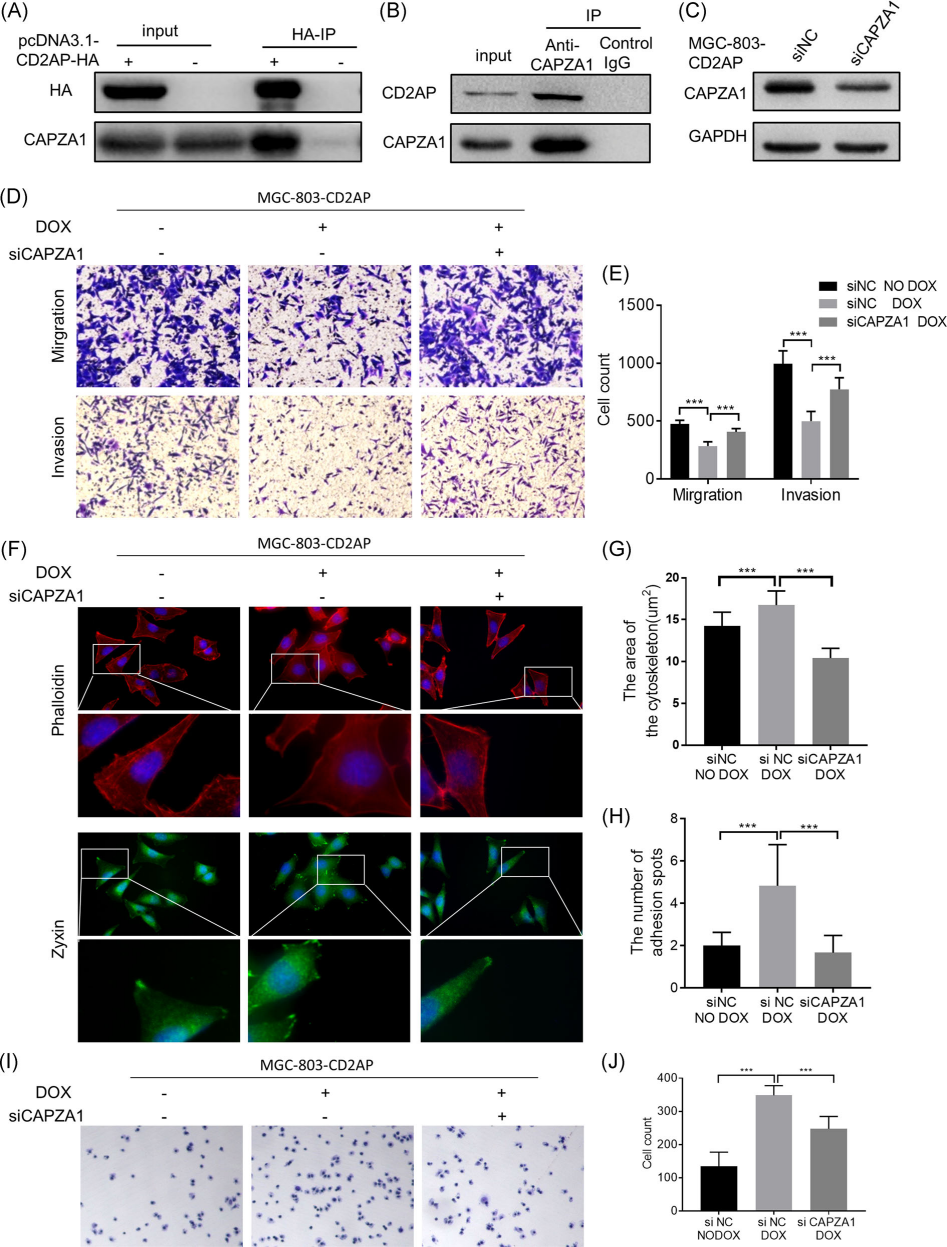
CD2AP interact with CAPZA1 mediated cellular adhesion and cytoskeleton assembly. A, Transfected MGC‐803 with pcDNA3.1‐CD2AP plasmid, empty plasmid transfected as control, Using Anti‐HA Magnetic Beads binding to HA‐ta, Western blot analysis show CD2AP interact with CAPZA1. B, Extracted MGC‐803 cell protein and incubated with anti‐capza1 antibody to form antigen‐antibody mixture, immunoprecipitation with Protein A/G Magnetic Beads, Western blot analysis show CD2AP interact with CAPZA1. C, MGC‐803‐CD2AP transfected with siNC or si‐CAPZA1 were analyzed by Western blot analysis with an antibody to CAPZA1. An antibody to GAPDH was used as an equal loading control. D and E, MGC‐803‐CD2AP cell transfected with si‐CAPZA1 and induced CD2AP expression by DOX. Depletion of CAPZA1 significantly rescues the ability of CD2AP to inhibit migration and invasion of GC cell (***P < .001). F‐H, MGC‐803‐CD2AP cell transfected with si‐CAPZA1 and induced CD2AP expression by DOX, Cell adhesion spots were labeled with zyxin antibody, the cytoskeleton were labeled with Phalloidin, depletion of CAPZA1 significantly rescue the ability of CD2AP which enhanced the assembly of cytoskeleton F‐actin and the number of focal adhesion spots (***P < .001). I and J, MGC‐803‐CD2AP cell transfected with si‐CAPZA1 and induced CD2AP expression by DOX. Depletion of CAPZA1 significantly rescues the ability of CD2AP to inhibit cell adhesion of GC cell (***P < .001). CD2AP, CD2‐associated protein; DOX, doxycycline; GC, gastric cancer [Color figure can be viewed at wileyonlinelibrary.com]

The F‐actin network is required for presynaptic assembly and for specificity‐determining adhesion spots.^28^ CAPZA1 can regulate the structural stability of F‐actin.^29^ Hence, we transfected the pcDNA3.1‐HA‐tagged CD2AP plasmid into MGC‐803 cells and immunoprecipitation with anti‐HA magnetic beads was carried out to determine the potential interaction between CD2AP and CAPZA1. Western blot analysis analyses showed that CD2AP could form a complex with CAPZA1 in the cells transfected with pcDNA3.1‐HA‐tagged CD2AP plasmid, but not in the cells transfected with pcDNA3.1 empty plasmid (Figure 6A). We also extracted MGC‐803 cell proteins, which were incubated with anti‐CAPZA1 antibody to form an antigen‐antibody mixture, and immunoprecipitation with Protein A/G magnetic beads was carried out. Western blot analysis analyses confirmed that CAPZA1 could form a complex with CD2AP in the GC cells (Figure 6B). These results suggested that CAPZA1 might act as a key mediator for CD2AP protein to promote cytoskeleton assembly and focal adhesion formation.

To examine how CAPZA1 influences the function of CD2AP of GC cells, we depleted CAPZA1 in MGC‐803‐CD2AP GC cells by siRNA (Figure 6C). Transwell assays showed that the depletion of CAPZA1 significantly rescued the ability of CD2AP to inhibit the migration and invasion of the GC cell line (Figure 6D,E). After depleting CAPZA1 in MGC‐803‐CD2AP GC cells, we induced the expression of CD2AP by DOX. Immunofluorescence analyses with phalloidin labeling for detection of cell adhesion spots showed that depletion of CAPZA1 significantly rescued the ability of CD2AP to enhance assembly of the F‐actin cytoskeleton and increased the number of focal adhesion spots (Figure 6F‐H). The cell adhesion assay indicated that the depletion of CAPZA1 significantly rescued the ability of CD2AP to enhance the adhesion ability of the GC cell line (Figure 6I,J).

## 4 DISCUSSION

GC metastasis involves infiltration of the surrounding tissues and blood vessels, transport of cells to the blood or lymph nodes, extravasation, and distant growth.^30^ Accumulating evidence indicates that DGC metastasis is closely related to alterations in the expression of cancer cell adhesion‐associated and cytoskeleton proteins. Cell‐to‐cell adhesion is critical for the maintenance of normal tissue morphogenesis and homeostasis,^6^ and in other cellular processes such as differentiation, survival, and migration, which are controlled by the activation of gene expression and signaling pathways.^31^ E‐cadherin, which is involved in the formation of cell‐cell adherent junctions that define the differentiation and proliferation of epithelial cells and suppression of invasion has been identified as an important tumor suppressor in GC.^32^, ^33^, ^34^, ^35^ An inactivating mutation of the *CDH1* gene or overexpression of its transcriptional repressor, alterations in the expression of microRNAs, deregulation of protein trafficking, and posttranslational modifications can all reduce the expression of E‐cadherin, which is closely related to the occurrence of DGC.^25^, ^26^, ^32^, ^36^, ^37^, ^38^

RhoA, a member of the Rho family, is a small GTPase that plays a fundamental role in regulating diverse cellular processes, including cell junction assembly, cell‐matrix adhesion, and cell migration. RhoGAP plays an important role in regulating the activation of RhoA.^39^, ^40^, ^41^, ^42^ In 2014, a high RhoA mutation rate and RhoGAP fusion were found in GC samples in the TCGA, and these mutations were present almost exclusively in DGC.^43^ The alteration in intercellular adhesion is an important feature of DGC.^44^ CD2AP is an important adhesion‐related adapter protein that plays a role in the formation of epithelial cell junctions.^10^ Xia et al^45^ reported that the association of CD2AP with the TGF‐3‐TRI complex activates both the p38 and the ERK signaling pathways, leading to a transient and reversible disruption of the blood‐testis barrier and Sertoli‐germ cell adhesion that facilitates germ cell migration. In the present study, we found that a low expression level of CD2AP in DGC was an independent protective factor for the prognosis of DGC. To our knowledge, this is the first clinicopathological study linking CD2AP to the outcome of the surgical treatment of GC. Furthermore, the downregulation of CD2AP significantly inhibited both the invasion and migration abilities of GC cells, which was consistent with the clinical data. Collectively, these results suggest that *CD2AP* might act as an oncogene during GC tumorigenesis, contributing new insight into the pathogenesis of GC metastasis.

CD2AP also acts as an adapter between membrane proteins and the actin cytoskeleton. The invasion and metastasis of cancer cells are closely related to cell migration, a process that involves actin filament dynamics regulated by various actin‐binding proteins.^46^ Cytoskeleton proteins play an important role in this process; for example, CAPZA1 interacts with cytoskeletal actin. Lee et al^21^ found that CAPZA1 is a marker for a good prognosis in GC and is associated with the decreased migration and invasion of cancer cells. Liu et al^47^ reported that Scinderin inhibited the formation of filopodia and decreased the expression of CDC42 along with GC cell migration. Srivatsan et al^48^ further indicated that knockdown of CD2AP in plasma cell‐like dendritic cells resulted in migration defects, and Zhao et al^49^ found that CD2AP functions synergistically to enhance the function of cortactin, which enhances branched actin filament formation and promotes cell spreading and migration in mice podocytes. Our results expand these roles of CD2AP as an important protective factor in GC.

The polymerization and depolymerization of F‐actin during tumor cell migration is an important process. We detected a direct interaction between CD2AP and the F‐actin capping protein CAPZA1, which regulates the structural stability of F ‐actin, which might explain the promotion of focal adhesion in GC cells by CD2AP overexpression. Indeed, several studies have shown that enhanced focal adhesion contributed to tumor progression by affecting the cytoarchitecture and motility,^27^, ^50^ which is consistent with our results. This could provide a mechanism for the inhibition of GC cell migration with a high expression of CD2AP.

## 5 CONCLUSIONS

In summary, our study illustrates an important function of CD2AP in the malignant behavior of GC. We demonstrated that CD2AP was expressed at a significantly low level in DGC and that a low level of CD2AP expression in GC tissue correlated with a poor prognosis. Furthermore, the downregulation of CD2AP expression enhanced GC metastasis by interacting with CAPZA1 to promote intercellular adhesion and to influence cell cytoskeleton. Therefore, CD2AP may represent a novel biomarker associated with a good prognosis for patients with GC.

## CONFLICT OF INTERESTS

The authors declare that there are no conflict of interests.

## Supporting information

Supporting information

Supporting information

Supporting information

Supporting information

## Data Availability

The data that support the findings of this study are available from the corresponding author upon reasonable request.
