# Supplementary figures and images for: CD2AP inhibits metastasis in gastric cancer by promoting cellular adhesion and cytoskeleton assembly
Source: Mol Carcinog. 2020 Jan 28;59(4):339–52. doi: 10.1002/mc.23158 (PMC7078920; doi:10.1002/mc.23158)

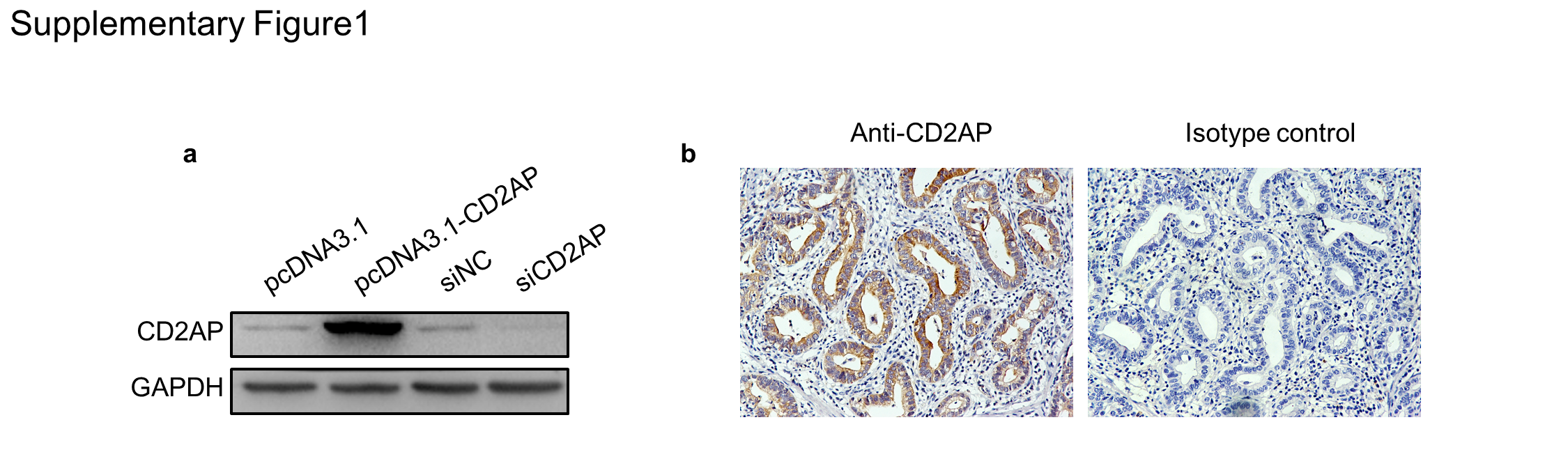

Supplement: Supplementary file 1 — Supporting information [file MC-59-339-s001.TIF]

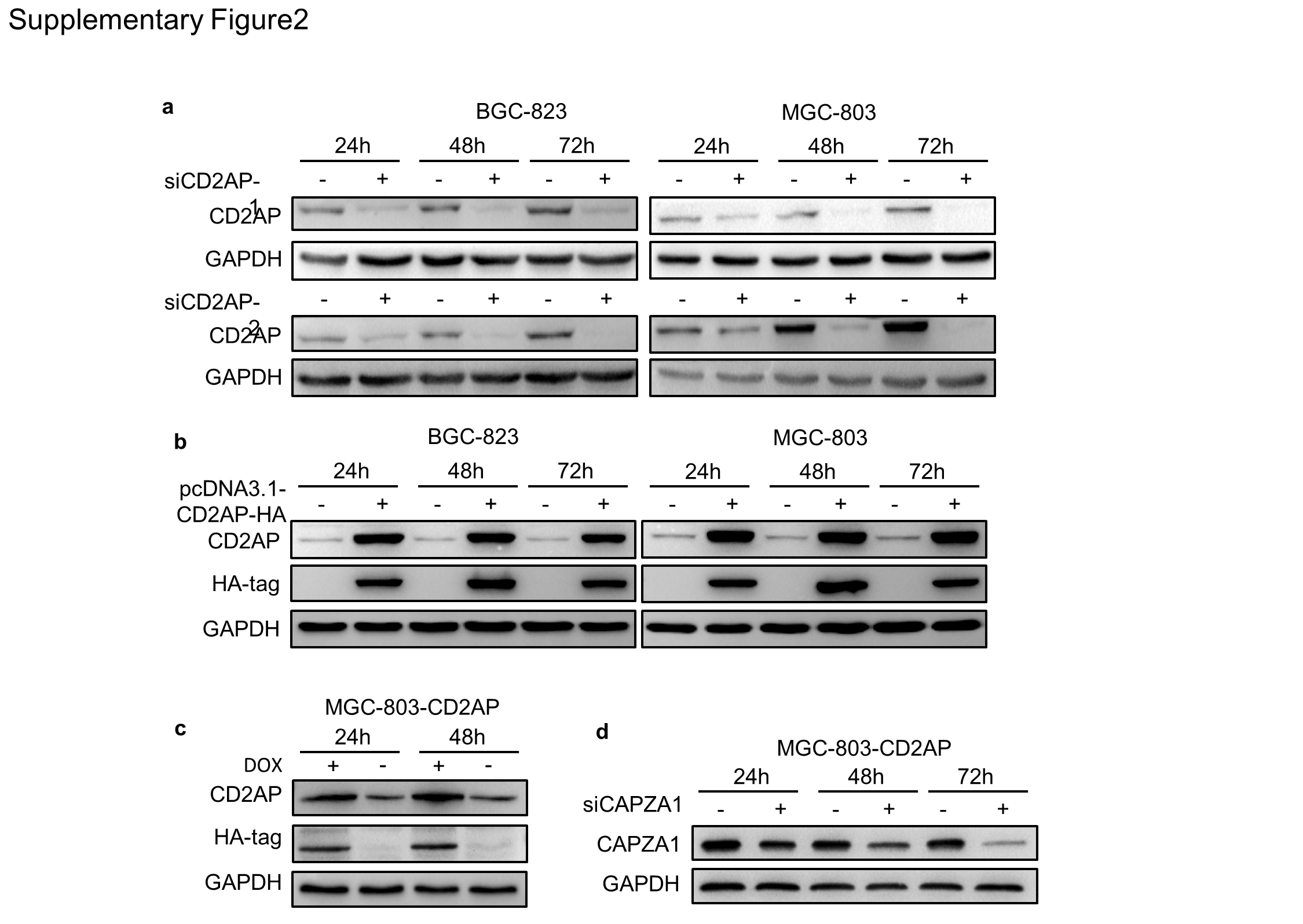

Supplement: Supplementary file 2 — Supporting information [file MC-59-339-s002.TIF]

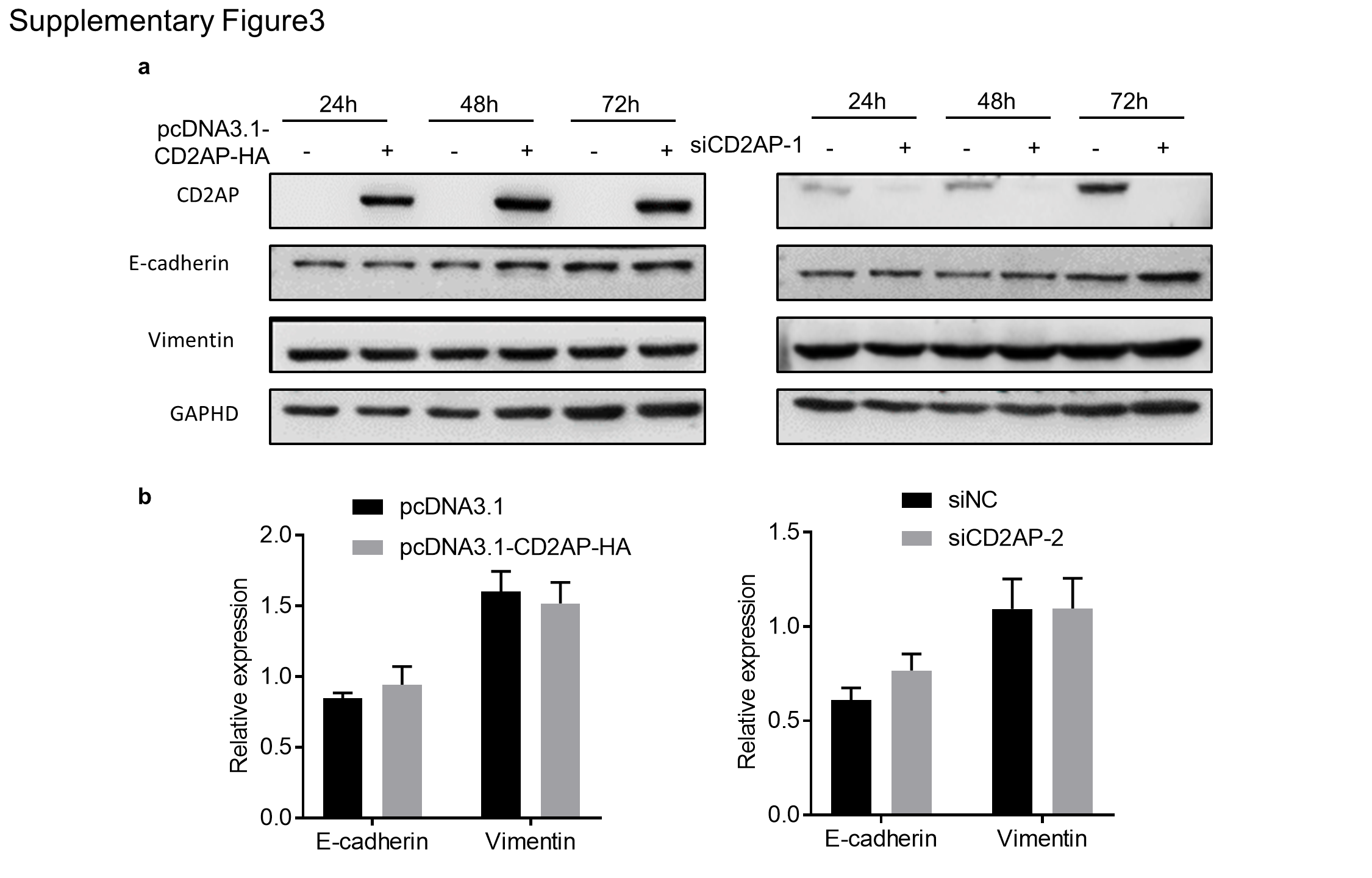

Supplement: Supplementary file 3 — Supporting information [file MC-59-339-s003.TIF]
