# Supplementary material for: CD2AP inhibits metastasis in gastric cancer by promoting cellular adhesion and cytoskeleton assembly
Source: Mol Carcinog. 2020 Jan 28;59(4):339–52. doi: 10.1002/mc.23158 (PMC7078920; doi:10.1002/mc.23158)
Supplement: Supplementary file 4 — Supporting information [file MC-59-339-s004.docx]

Supplementary Figure1 Verify the specifIcity of CD2AP antibody.

(a)BGC-823 cells transfected with pcDNA3.1, pcDNA3.1-CD2AP-HA, siNC and si- CD2AP, Western blot by immunoblotting with an antibody to CD2AP. An antibody to GAPDH was used as an equal loading control. (b) Immunohistochemistry detect gastric cancer tissue by antibody to CD2AP, Ig G was used as an equal loading control.

Supplementary Figure2 Overexpression or knockdown of CD2AP and CPAZA1 in gastric cancer cell lines.

(a) The specificity of the two different CD2MGC-803 transfected with empty (siNC) or CD2AP si-RNAs (si-CD2AP-1, si-CD2AP-2) were analyzed by Western blotting with an antibody to CD2AP. An antibody to GAPDH was used as an equal loading control. (b) BGC-823,MGC-803 transfected with pcDNA3.1 and pcDNA3.1-CD2AP-HA upregulated CD2AP gene expression and were analyzed by Western blotting with an antibody to CD2AP. An antibody to GAPDH was used as an equal loading control (c) MGC-803-CD2AP induction CD2AP expression by 1ug/ml DOX, and were analyzed by Western blotting with an antibody to CD2AP. An antibody to GAPDH was used as an equal loading control. (d) MGC-803-CD2AP transfected with siNC or si-CAPZA1 were analyzed by Western blotting with an antibody to CAPZA1. An antibody to GAPDH was used as an equal loading control.

Supplementary Figure3 CD2AP does not inﬂuences gastric cancer cells epithelial–mesenchymal transition.

(a) Western blotting analysis of E‑cadherin and Vimentin protein levels in BGC-823 cells transfected with pcDNA‑CD2AP-HA or si‑CD2AP-1. antibody GAPDH was used as an internal control. (b) The change of E-cadherin and Vimentin in cells overexpressing and knocking down CD2AP is not significant(P>0.05).
